# Effects of Sleep on Language and Motor Consolidation: Evidence of Domain General and Specific Mechanisms

**DOI:** 10.1162/nol_a_00060

**Published:** 2022-02-16

**Authors:** Dafna Ben-Zion, Ella Gabitov, Anat Prior, Tali Bitan

**Affiliations:** Department of Learning Disabilities, University of Haifa, Haifa, Israel; Edmond J. Safra Brain Research Center for the Study of Learning Disabilities, University of Haifa, Haifa, Israel; Institute of Information Processing and Decision Making, University of Haifa, Haifa, Israel; The Integrated Brain and Behavior Research Center (IBBRC), University of Haifa, Haifa, Israel; McConnell Brain Imaging Center, Montreal Neurological Institute, McGill University, Montreal, Quebec, Canada; Department of Psychology, University of Haifa, Haifa, Israel; Department of Speech Language Pathology, University of Toronto, Toronto, Ontario, Canada

**Keywords:** consolidation, language, morphology, sleep, motor, grammar

## Abstract

The current study explores the effects of time and sleep on the consolidation of a novel language learning task containing both item-specific knowledge and the extraction of grammatical regularities. We also compare consolidation effects in language and motor sequence learning tasks, to ask whether consolidation mechanisms are domain general. Young adults learned to apply plural inflections to novel words based on morphophonological rules embedded in the input, and learned to type a motor sequence using a keyboard. Participants were randomly assigned into one of two groups, practicing each task during either the morning or evening hours. Both groups were retested 12 and 24 hours post-training. Performance on frequent trained items in the language task stabilized only following sleep, consistent with a hippocampal mechanism for item-specific learning. However, regularity extraction, indicated by generalization to untrained items in the linguistic task, as well as performance on motor sequence learning, improved 24 hours post-training, irrespective of the timing of sleep. This consolidation process is consistent with a frontostriatal skill-learning mechanism, common across the language and motor domains. This conclusion is further reinforced by cross-domain correlations at the individual level between improvement across 24 hours in the motor task and in the low-frequency trained items in the linguistic task, which involve regularity extraction. Taken together, our results at the group and individual levels suggest that some aspects of consolidation are shared across the motor and language domains, and more specifically, between motor sequence learning and grammar learning.

## INTRODUCTION

Creating durable and accessible knowledge is essential to second language learning, as it is for learning other cognitive and motor skills. Memory research has identified consolidation as a key process in establishing such long-term representations (Dudai et al., 2015). The role of sleep in memory consolidation has been investigated in declarative / episodic memories (Mölle et al., 2011; Plihal & Born, 1997; Wilhelm et al., 2011) and in procedural motor sequence learning (Brashers-Krug et al., 1996; Korman et al., 2003, 2007; Robertson, Pascual-Leone, & Miall, 2004; Stickgold, 2005), but the factors that determine when sleep plays a critical role in consolidation are still under debate (Cordi & Rasch, 2021; Diekelmann & Born, 2010; King et al., 2017; Schmidt et al., 2020). Sleep dependent consolidation in language learning has been studied mainly in vocabulary learning (Davis & Gaskell, 2009; Dumay & Gaskell, 2007; James et al., 2017; Schreiner & Rasch, 2017), which relies on item-specific, declarative hippocampal mechanisms. Grammar learning, in contrast, is suggested to rely to a greater extent on procedural non-hippocampal learning mechanisms, which may be akin to motor skill learning (Hedenius et al., 2011; Ullman, 2015). In the current study, we examine the effect of sleep on the consolidation of a morphological learning task that enables us to study item-specific knowledge, the extraction of grammatical regularities, and the interactions between them. The study also compares the effect of sleep on language learning to its effect on motor sequence learning, to identify the shared and distinct factors that affect consolidation in both domains. Finally, we ask to what extent individual differences determine consolidation across the language and motor domains.

### Memory Consolidation

*Memory consolidation* is a process that transforms new and initially labile memories into more stable representations that become integrated into the network of pre-existing long-term memories (Diekelmann & Born, 2010). This process is time-dependent and can also be sleep-dependent (Dudai et al., 2015). Memory consolidation is considered sleep-dependent when a period of post-learning sleep enhances performance or qualitatively changes the representation of information compared with a wake interval of equal length (Korman et al., 2007; Rasch et al., 2007; Tucker et al., 2006; Walker et al., 2003). While evidence for sleep-dependent consolidation was shown in both hippocampal and non-hippocampal-dependent tasks, the type of learning may have an effect on the pattern of consolidation (Diekelmann et al., 2009; Diekelmann & Born, 2010; Song, 2009).

In the context of hippocampal-based learning, the complementary learning system (CLS) framework (McClelland, 2013; McClelland et al., 1995) and the active system consolidation hypothesis, suggest that memories initially encoded in parallel in neocortical networks and in the hippocampus are repeatedly reactivated in the hippocampus during sleep, and thereby become gradually redistributed within the neocortex (Diekelmann & Born, 2010). The reorganization in the neocortex during consolidation enables the generation of new associations through the extraction of overlapping features from separate events and thereby eventually facilitates novel inferences that afford generalization and insight (Diekelmann & Born, 2010).

Consolidation of non-hippocampal learning, including perceptual learning (Censor et al., 2006; Fenn et al., 2003) and motor skill acquisition (described in detail below), can also benefit from sleep (King et al., 2017; Korman et al., 2003; Walker & Stickgold, 2004). Circuit-level redistribution of experience-dependent representational information over time can occur in tasks that can be acquired independently of the hippocampus and the medial temporal lobe (Dudai et al., 2015). For example, the synaptic homeostasis hypothesis (Tononi & Cirelli, 2006, 2014) suggests that information encoding during wakefulness leads to a net increase in synaptic strength in the brain. Sleep then serves to globally downscale synaptic strength. As a result of this renormalization process, weakly activated synapses become virtually ineffective after sleep, whereas strongly activated synapses are preserved and may consolidate (Crick & Mitchison, 1983; Tononi & Cirelli, 2006). Nevertheless, there is also evidence that corticostriatal and hippocampal-dependent memory systems do not act independently, but rather interact during consolidation (Albouy et al., 2015; Coynel et al., 2010; Debas et al., 2014), so that even tasks that do not depend on the hippocampal system during initial phases of acquisition, may show hippocampal-based sleep dependent consolidation (King et al., 2017; Klinzing et al., 2019; Sawangjit et al., 2018).

Behavioral changes following consolidation processes are expressed as enhancement in performance or as stabilization (Censor et al., 2012; Diekelmann & Born, 2010; Ellenbogen et al., 2007; Ellenbogen, Hulbert et al., 2006; Fischer et al., 2006; Korman et al., 2007; Rasch & Born, 2013; Wagner et al., 2004). While there is a debate on whether sleep-related stabilization and offline gains represent similar or different processes, there is evidence that both are related to an active role of sleep in consolidation (Diekelmann & Born, 2010).

### Consolidation in Language Learning

To date, most research on sleep-dependent consolidation in language learning focused on vocabulary learning (Gais et al., 2006; Henderson et al., 2012; James et al., 2017; Landi et al., 2018), which is typically associated with item-specific knowledge and episodic learning. The adaptation of the CLS framework to word learning (Davis & Gaskell, 2009) suggests that a newly learned word is initially stored as a distinct episodic trace relying on the hippocampus, but following an offline consolidation period it becomes integrated with the existing lexicon in neocortical long-term semantic memory. Support for this notion comes from studies showing that the integration of new linguistic knowledge into the existing lexicon is sleep-dependent (Dumay & Gaskell, 2007; Henderson et al., 2013; Tamminen et al., 2010). To wit, performance on recall tests of the newly learned vocabulary, 12 to 24 hours post-training, shows a stabilizing effect when sleep is afforded immediately after training compared to an equivalent period of wakefulness (Gais et al., 2006; Tamminen et al., 2010). Moreover, other studies even reported offline gains, measured as an increase in the number of recalled words or a reduction in reaction time following a night’s sleep compared to performance immediately after training (Dumay & Gaskell, 2007; Heim et al., 2017; Henderson et al., 2012).

In contrast to vocabulary, grammar learning is suggested to rely to a greater extent on procedural skill learning mechanisms associated with frontostriatal brain regions (Hedenius et al., 2011; Ullman, 2015). The term *procedural learning* is often associated with implicit learning, or lack of awareness, which is not necessarily the case for regularity extraction in the current study, and in grammar learning more generally. Therefore, we avoid using this term here, and instead we refer to *skill learning*. Given the differential role that sleep may play in the consolidation of hippocampal- vs. non-hippocampal-dependent learning (Diekelmann & Born, 2010), it is important to examine the role of sleep in the consolidation of grammar learning. Studies examining the effect of sleep on statistical or serial order learning outside the language domain report contradicting findings, as some found that sleep increased recognition of new rule-based sequences (Durrant et al., 2011; Lerner & Gluck, 2019; Nieuwenhuis et al., 2013), but others found no benefit (Szmalec et al., 2012).

Very few studies investigated sleep dependent consolidation following learning of grammatical and morphological regularities in language (Batterink et al., 2014; Kim & Fenn, 2020; Mirković & Gaskell, 2016; Tamminen et al., 2010), and here too there are contradicting results. Two studies found no benefit of sleep for extracting morphological regularities in a novel language using a short nap (Mirković & Gaskell, 2016) or nocturnal sleep deprivation (Tamminen et al., 2020). Only one study found an association between the extraction of grammatical regularities and sleep (Batterink et al., 2014). In this study, participants’ sleep was monitored during an afternoon nap that followed training on new syntactic rules. The results showed that the amounts of slow-wave and rapid-eye-movement sleep predicted participants’ sensitivity to implicit regularities embedded in the stimuli (Batterink et al., 2014). However, because only one group of participants was examined, the effect of sleep cannot be compared to the effect of wake. A recent study examined the effect of explicit awareness on sleep-dependent consolidation following syntactic rule learning in a second language (Kim & Fenn, 2020). They found that only those participants who were aware of the grammar rules at the end of training improved their performance following sleep, suggesting that the benefit of sleep for extraction of grammatical regularities during consolidation depends on explicit awareness.

Given these mixed findings, the current study explores the effect of sleep on consolidation in a language task that affords both item-specific learning and extraction of morphological regularities. These two aspects of language learning may involve different consolidation mechanisms and thus may be differentially affected by sleep. The effects of sleep on consolidation in the language task will also be compared to these effects in a motor sequence learning task in order to examine domain generality of consolidation mechanisms.

### Consolidation in Motor Learning

Motor skill learning is one of the most studied domains in relation to sleep dependent consolidation. It is broadly accepted that motor learning takes place not only online (i.e., during task practice) but also offline (i.e., between training sessions) in the absence of any further practice (Doyon et al., 2018; Karni et al., 1998; King et al., 2017; Nettersheim et al., 2015; Schmidt et al., 2020). However, the role of sleep in such offline processes is still hotly contested.

Whereas sleep is generally beneficial in consolidating skills, it might be critical only in some motor tasks, particularly those requiring movement sequence learning and generating new movement routines (Debas et al., 2010, 2014; Korman et al., 2007; see also recent reviews by Cordi & Rasch, 2021; Dudai et al., 2015; Hu et al., 2020; King et al., 2017; Rasch & Born, 2013). This beneficial effect of sleep can be reflected behaviorally either by enhancement or stabilizing the skill levels that were achieved by the end of training (Maltry et al., 2020; Robertson, 2012). For example, improved performance after post-training sleep was documented in finger motor sequence tasks (Fischer et al., 2002, 2005; Korman et al., 2007; Walker et al., 2002).

Recently, several key findings supporting the idea of sleep-dependent consolidation of motor sequence knowledge have been called into question (for the results of a meta-analysis, see Pan & Rickard, 2015, and a review by Cellini, 2017). Some studies suggest that sleep does not improve performance of the trained motor sequence (Brawn et al., 2010; Nettersheim et al., 2015; Rickard et al., 2008), but rather facilitates recovery of a previously attained skill level after it has been degraded during the post-training wake interval (Brawn et al., 2010). Others propose that the simple passage of time and time spent in sleep may facilitate different aspects of motor sequence knowledge (King et al., 2017), and that participants’ awareness of the learned sequence may determine the necessity of sleep in consolidating that sequence (Robertson, Pascual-Leone, & Press, 2004).

### Associations Between Language and Motor Consolidation

In the current study, we directly compare the effect of sleep on consolidation of motor and language tasks and ask whether consolidation processes might be shared across domains. The idea of shared learning mechanisms across domains is supported by the notion that motor sequence learning and language grammar learning may both rely on procedural learning (Hedenius et al., 2011; Ullman, 2015). Thus, although the elements of each task are different, i.e., morphemes versus finger movements, in both tasks participants learn to link these elements based on regular patterns. Several lines of evidence lead to the hypothesis of a shared consolidation process in motor sequence and grammar learning, including common neural substrates and learning mechanisms across domains, as well as shared properties of consolidation.

First, there is evidence that both motor sequence learning and language learning rely to some extent on similar brain areas, including the supplementary motor area (SMA) and the basal ganglia. The SMA is involved in the integration of sequential elements into unified representations, in both motor (Cona & Semenza, 2017; Orban et al., 2010; Wymbs & Grafton, 2013) and language tasks (Ferstl et al., 2008; Hertrich et al., 2016; Nakai et al., 1999; Needle et al., 2015; Segaert et al., 2012), and specifically in the sequential integration of linguistic elements into higher order representations (Cona & Semenza, 2017). The basal ganglia are also involved in motor sequence learning (Albouy et al., 2008; Gabitov et al., 2015; Hikosaka et al., 2002; Lehéricy et al., 2005; Penhune & Steele, 2012) and language learning tasks (Chan et al., 2013; Nevat et al., 2017; Ullman & Pierpont, 2005).

Evidence for these shared mechanisms also comes from children with developmental language disorder showing deficits in motor serial reaction time tasks, and other tasks associated with procedural learning (see meta-analysis by Lum et al., 2014). These findings are the basis for the procedural deficit hypothesis (Lum et al., 2014; Ullman & Pierpont, 2005), which suggests that language and grammar deficits in developmental language disorders are linked to a more general deficit in procedural learning mechanisms, rooted in the SMA and frontostriatal circuits (Ullman & Pierpont, 2005). However, a recent study states that the implicit learning tasks that are often used to test procedural learning capacities have low reliability (West et al., 2017), raising some questions regarding this interpretation. Similarly, patients with Parkinson’s disease, which is characterized by motor control deficits, show impairments in morphological processing (Eyigoz et al., 2020), again suggesting a shared neural substrate for learning and processing in the two domains.

Second, consolidation of both motor and language learning are associated with similar sleep mechanisms. Specifically, time spent in stage 2 sleep and specific characteristics of stage 2 spindles are associated with improved performance after sleep in motor tasks (Morin et al., 2008; Peters et al., 2008), a vocabulary learning task (Mirković & Gaskell, 2016), and other hippocampal-dependent episodic memory tasks (Mednick et al., 2013; Schabus et al., 2004). Finally, individual differences in various participant characteristics have been linked to consolidation in different domains, raising the possibility of a shared underlying mechanism. For example, age is a factor influencing consolidation—adolescents showed a smaller consolidation effect than young adults in vocabulary learning (Landi et al., 2018), and older adults showed reduced consolidation of motor sequence learning following sleep than did younger adults (Spencer et al., 2007; see also Wilhelm et al., 2008, 2013). Similarly, general cognitive abilities such as working memory or intelligence affect consolidation in episodic memory tasks (Fenn & Hambrick, 2012, 2015). Finally, individual differences in non-motor domains, such as those reflected in attention deficit disorder or dyslexia, are also linked to reduced consolidation in motor learning (Adi-Japha et al., 2011; Needle et al., 2015; Wilhelm et al., 2012).

Taken together, these findings suggest that consolidation processes in motor sequence learning and language learning might be governed by similar underlying mechanisms. However, a recent study that directly examined consolidation of a Hebb repetition task (which bears similarities to language learning) and of a motor serial reaction time task, though without manipulation of sleep, did not find any cross-domain correlations (Henderson & Warmington, 2017). This finding calls into question the idea of a general consolidation ability. The current study directly addresses the questions of sleep dependency and domain generality of consolidation, by comparing the effect of sleep on morphological learning and on motor sequence learning and examining the correlations between the two.

### Objectives of the Current Study

In the current study, we investigate the effect of sleep on consolidation of learning morphological regularities in a novel language and compare it to the effect of sleep on motor sequence learning, in a typical adult population. For the motor learning, we use a computerized version (Gabitov et al., 2019) of a finger sequence learning task that has previously been shown to be affected by sleep (Fischer et al., 2002, 2005; Karni et al., 1998; Korman et al., 2007). For the language task, we use an artificial language paradigm in which participants learn to apply plural inflections to novel words based on morphophonological rules embedded in the input, adapted from previous studies (Ben Zion et al., 2019; Nevat et al., 2017, 2018). Performance in early stages of learning in a similar task was previously shown to rely on frontostriatal areas (Nevat et al., 2017).

We manipulated the timing of training so that one group was trained in the morning (wake-first) and the other group was trained in the evening (sleep-first), and both groups were tested 12 and 24 hours post-training, to determine the effect of sleep versus the passage of time. Participants performed the language and motor tasks with the same schedule. This design allowed us to probe the existence and characteristics of the posited general consolidation ability in two complementary ways. First, at the group level, we examined the effect of sleep on consolidation in both the motor and language domains in the same group of participants. Second, we examined whether individuals’ consolidation gains in the two tasks are correlated, to test whether the ability to consolidate newly encoded knowledge relies on learning mechanisms that are common to both domains.

The current version of the artificial language task was constructed such that it allows us to differentiate, to some extent, between item-specific learning and the extraction of regularities, i.e., the learning of the grammar. To this end, we manipulated the frequency of presentations of items during training. Hence, performance on high frequency items reflects processes of item-specific learning more than performance on low frequency items, which relies on both item-specific and regularity extraction components. Previous studies also support this differentiation between high and low frequency presentation, by demonstrating that the consolidation of items tends to benefit differently from sleep, dependent on the strength of the initial encoding (Denis et al., 2020; Diekelmann et al., 2009; Stickgold, 2009). In contrast to performance on trained items, which partially reflects item specific learning, the generalization to untrained items was solely dependent on the ability to extract the regularity from the trained input.

We predicted that sleep will promote consolidation in both the language and motor tasks, but the benefit of sleep may be reflected differently for each task. In the language task, we predicted that sleep will result in the stabilization of item-specific knowledge (evident by performance on high frequency items), as was previously evident in vocabulary learning and episodic memory tasks (Gais et al., 2006; Payne et al., 2012; Peiffer et al., 2020; Tamminen et al., 2010). Based on findings of sleep related offline gains in motor sequence learning tasks (Fischer et al., 2002, 2005; Karni et al., 1998; Korman et al., 2007) and the effect of sleep on grammar learning (Batterink et al., 2014), we predicted that sleep will improve performance in the motor task and in linguistic tasks that rely on the extraction of regularities, namely low frequency trained items and generalization to untrained items. We also expected this aspect of grammar learning to be correlated with motor sequence learning.

## MATERIALS AND METHODS

### Participants

Forty-four healthy young adults participated in the study. Of these, 38 (18–33 years old; mean = 24.295, *SD* = 3.32; 29 women) are included in the analysis of the language learning task, and of them 36 participants are also included in the analysis of the motor sequence learning task. Three participants dropped out due to technical and personal issues, and two participants were excluded due to low performance (1.5 *SD* below the mean) in two reading screening tests. Each participant was randomly assigned to either the morning *wake-first* group (*n* = 20) or the evening *sleep-first* group (*n* = 18). The group assignment for each participant was the same for the motor and language tasks. Nineteen and 17 participants from the wake-first and sleep-first groups, respectively, completed both the linguistic and motor learning tasks with a minimum of 2 weeks apart. The order of the tasks was counterbalanced across participants so that half of the participants in each group participated in the language task first, and half in the motor task first.

All participants were native Hebrew speakers and spoke at least one other language (English) as a foreign language. They were right-handed (self-report) and had normal or corrected-to-normal vision and no hearing deficits. The screening procedure included self-report of no history of neurological or psychiatric illness, no learning disability or attention disorder, no addiction to alcohol (no more than one alcoholic beverage a day), and being a non-smoker. In addition, to ensure good quality of sleep, exclusionary criteria included the use of medication that affects sleep, mid-day naps, pregnancy, working night shifts, trans-Atlantic trips within 3 months prior to the study, drinking more than 3 caffeinated beverages per day, and obesity (BMI > 30; group mean = 22.58; *SD* = 2.45). Sleep disorders were ruled out by the Mini Sleep Questionnaire (Natale et al., 2014; Zoomer et al., 1985), and circadian tendency was measured using the Hebrew version of the Morningness-Eveningness Questionnaire (Horne & Ostberg, 1976); both showed no significant differences between the groups. Participants maintained between 6 and 9 hours of proper nocturnal sleep and abstained from caffeinated and alcoholic drinks 24 hours prior to the experiment and during the experiment itself (which lasted 36 hours), as reported in a sleep log.

Participants’ normal reading level was confirmed by two screening tests: the one-minute word reading test and the one-minute pseudoword reading test (Shatil, 1995, 1997). In these tests, participants read a list of pointed words or pseudowords as quickly and as accurately as possible within 1 minute, and the number of correct items was counted. Only two participants met the exclusion criterion of more than 1.5 *SD* below the average of our local norms (Weiss et al., 2015) in both tests, and were excluded.

In order to make sure the two groups did not differ on phonological and morphological awareness as well as on working memory ability, the following tests were conducted: (1) the Phoneme Deletion Test for Pseudowords (Ben-Dror & Shani, 1996); (2) morphological awareness was tested using two production tests: a word-inflection task (Cohen-Mimran, 2009) and a word-derivation task (Raz-Salzburg & Ravid, 2009); and (3) short term and working memory were assessed using the digit span task from the Wechsler Adult Intelligence Scale (Wechsler, 1955) and were collapsed to a normalized working memory *z* score. The two groups did not differ significantly on any of these measures (Table 1).

**Table 1.** Mean score and standard deviation per group in screening tests

**Screening test**		**Wake-first group**		**Sleep-first group**	
		mean	*SD*	mean	*SD*
**Word reading test**	Words per minute	107.1	18.6	104.5	17.6
**Pseudoword reading test**	Nonwords per minute	56.5	11.9	54.7	12.4
**Phoneme Deletion Test for Pseudowords**	Accuracy	92.2%	11.7%	92.7%	8.0%
**Inflectional morphology**	Accuracy	87.7%	9.3%	88.0%	11.1%
**Derivational morphology**	Accuracy	96.2%	3.2%	96.6%	3.8%
**Working memory**	*z* score	10.4	2.7	11.6	2.9

This study was reviewed and approved by an ethics committee. All participants gave written informed consent and received financial compensation.

### Design and Experimental Procedure

**Figure 1.**
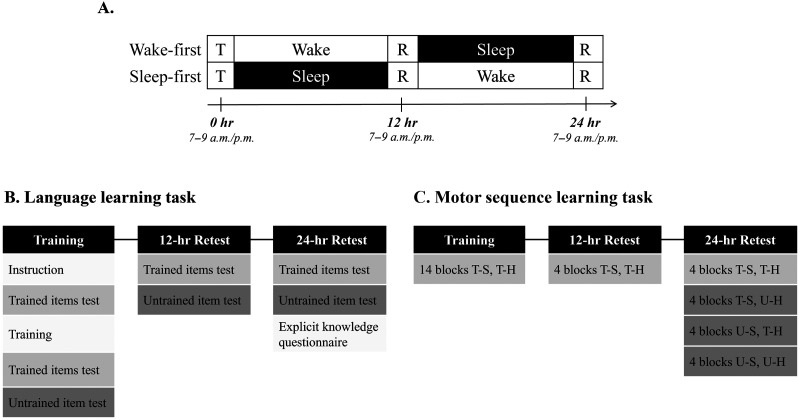
Overall design of the experiment. (A) The wake-first group performed training (T) of either the motor or the linguistic learning task, during the morning between 7 and 9 a.m., retested (R) after 12 hours of wakefulness, and retested again after a period of sleep. The sleep-first group preformed training during the evening, retested following a period of sleep, and retested again after an additional 12 hours. (B) Language learning task design, consisting of three sessions. (C) Motor sequence learning task consisting of three sessions. T-S refers to Trained Sequence while U-S refers to Untrained Sequence; T-H refers to Trained Hand while U-H refers to Untrained Hand.

The study consisted of two experimental tasks: (1) language learning, and (2) motor sequence learning, conducted on separate days with at least 2 weeks apart. The experimental protocol for each task was composed of three sessions separated by 12 hours. First, a screening session was administered (usually conducted concurrently with the first experimental session), in which baseline parameters were measured. Next, participants learned either to generate a motor sequence or to inflect items according to a new linguistic regularity. For both tasks, the first session included training and the second and third sessions included only retests. For the motor task, generalization of the learned skill was measured only at the end of the last session, whereas for the linguistic task, generalization was tested at the end of each session. Morning and evening sessions were conducted between 7–9 a.m. and 7–9 p.m., respectively (Figure 1A).

#### Language learning task

The task and stimuli were adapted from previous studies (Ben Zion et al., 2019; Nevat et al., 2017, 2018). The trained items consisted of 36 novel words, which were aurally presented together with pictures of the objects they refer to (e.g., real objects like an apple). All items consisted of two syllables (CVCVC) in their singular form (the stem). Plural forms were created by applying one of three possible (VC) suffixes to the stem (-*an*, -*esh*, -*ur*); each suffix was applied to one third of the items (12 items).

Pairings of stems and suffixes were determined by the last two phonemes of the stem, the phonological cue, such that each suffix was associated with two phonological cues. For example, stems ending with /oz/ and with /ap/ took the suffix -*an*; thus, the plural for *kut**oz*** was *kutoz**an*** and the plural for *nif**ap*** was *nifap**an*** (see full details in Table 2). However, 6 trained items (2 of each suffix) did not follow these rules and were included in order to increase the difficulty of learning the regularities, thus mimicking the inconsistency of natural languages. These *exception* words took a different suffix from the one predicted by the words’ phonological cue. For example, although most words ending with the cue /oz/ received the suffix -*an*, the stem *nuboz* received the suffix -*esh* forming the word *nuboz**esh***, which did not adhere to the general inflection rule. These exception words were removed from the analysis because previous studies found that these words show lower accuracy than consistent words (Nevat et al., 2017), and their small number does not allow for a separate analysis.

**Table 2.** Trained words

	Suffix -*an*		Suffix -*esh*		Suffix -*ur*	
High frequency	laloz	nifap	motsid	sibaf	libok	pikis
	refoz	tuvap	rilid	tegaf	torok	fazis
	bomoz	datsap	zutid	dumaf	shuzok	reshis
Low frequency	gishoz	venap	lebid	bodaf	navok	molis
	kutoz	sogap	panid	gazaf	delok	vusis
Exceptions	gukid	lagis	nuboz	zelok	potaf	filap

*Note*. List of trained items, presented by suffix and frequency (high and low). Exception items, presented at a low frequency, received unpredictable suffixes and were removed from the analysis.

During training, half of the items (18; 6 of each suffix, all with consistent cues) were presented 3 times per block (high-frequency items), while the other half were presented only once per block (low-frequency items). One third (6) of the low-frequency items were *exception* words (Table 2). Since the exception words were excluded, the analysis included fewer low frequency than high frequency words (12 and 18 respectively). Frequency was manipulated in order to differentiate between item-specific learning and regularity extraction. Specifically, in the current design, since both high and low frequency items share the same regularities, the differences between them can only be attributed to item-specific learning, which plays a more prominent role in high frequency items.

Generalization of the morphological regularity was tested by inflecting untrained items. At the end of each session, participants were also tested on the inflection of 30 unique untrained (new) items, resulting in a total of 90 untrained items across all sessions. In each generalization test of 30 items, there were 5 words containing each of the 6 phonological cues, resulting in 10 items receiving each of the three suffixes.

The language task was performed over three sessions: (1) the first session, which included the instruction block, the pre-training trained items test, three blocks of training including feedback, the post-training trained items test, and finally a generalization test on untrained items; (2) the 12-hr retest session, which consisted of a trained items test and a generalization test; and (3) the 24-hr retest session, which consisted of trained items and generalization tests, and an explicit rule knowledge questionnaire (Figure 1B).

**Figure 2.**
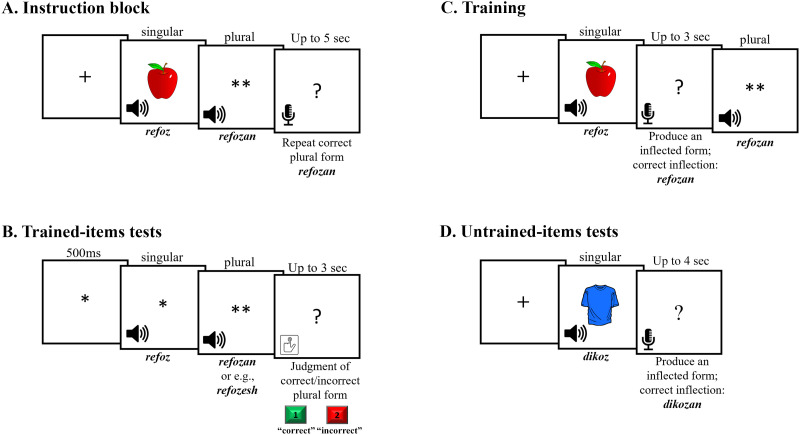
Design of trials. (A) Instruction block: each item was presented once, together with the picture that refers to its meaning. (B) Trained-items tests: each item was tested once and required the judgment of correctly and incorrectly inflected plural forms. (C) Training: participants produced the inflected forms of the trained items, receiving feedback. (D) Untrained-items tests: participants inflected untrained items from their singular to their plural form.

In the instruction block, each of the 36 training items was presented once. Upon key press, the singular form was presented aurally together with an image of a real object to be learned as its referent on the screen. The singular form was followed by a visual cue consisting of two asterisks (**), indicating the plural form of the word would soon be presented. The plural form was then presented aurally, followed by the presentation of a question mark, indicating that participants were to repeat the plural form they had just heard. The question mark remained on the screen for a maximal duration of 5 sec, or until a vocal response was detected (Figure 2A).

The trained-item test required the judgment of correctly and incorrectly inflected plural forms that were presented both before and after training in the first session, and in each of the following two sessions (4 trained-item tests overall). Each of the 36 trained items was presented once in each test. During the test, the singular form was aurally presented (without its picture) followed by an aurally presented plural form of the same word. Participants were instructed to press “1” on a standard keyboard if the plural form was correct, and “2” otherwise. They were given 3 sec to respond (Figure 2B). In each test, half of the presented 36 plural forms were correct, and half were incorrect. Incorrect inflections were created by adding one of the other suffixes to the stem. Across sessions, each participant was presented with all different incorrectly affixed forms in a random order.

Training took place in the first session (Figure 1B). During training, participants heard the singular form accompanied by its picture and attempted to produce the plural form. The correct plural form of the word was then presented aurally, as feedback (Figure 2C). The training session consisted of three blocks, separated by breaks. In each block half of the items, *low-frequency* items, were presented once (18 trials), and half, *high frequency* items, were presented 3 times (54 trials), resulting in a total of 3 or 9 presentations per word, respectively (Table 2). The order of items within each block was randomized.

In the untrained-items tests (generalization task), participants were asked to inflect 30 untrained items from their singular to their plural form (Figure 2D). Words were presented in a randomized order. The untrained-items test was presented at the end of each session (Figure 1B), for a total of three times.

At the end of the third linguistic session (in the 24-hr retest), participants answered a questionnaire assessing their explicit knowledge of the inflection regularity. Participants were asked to write down which suffixes they remember, and to explicate what rule guided them in inflecting singular stems to their plural form.

#### Motor sequence learning task

**Figure 3.**
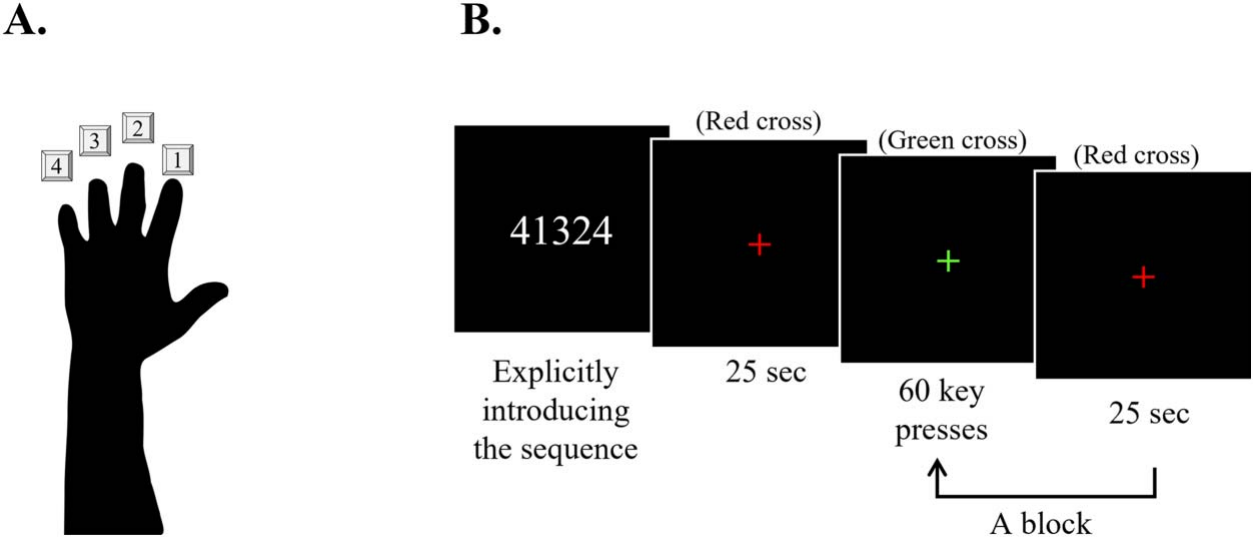
Motor sequence learning task setup and trials. (A) Illustration depicting the mapping of digits to numbers. (B) Each session began with an explicit introduction of the sequence, followed by 14 training blocks (indicated by a green cross). The blocks comprise 60 key presses—the code and the time of each pressed key are registered—and are separated by rest periods of 25 sec (red cross).

Participants were trained to perform accurately and repeatedly a 5-element sequence by tapping a 4-key response pad with their left (non-dominant) hand (Figure 3A). This computerized version of the sequential finger-tapping task, adapted from Karni et al. (Karni, 1995; Karni et al., 1998), has been widely used to study mnemonic processes underlying learning of a new motor skill (Albouy et al., 2015; Censor et al., 2010; Gabitov et al., 2019; Gal et al., 2019; King et al., 2017; Walker et al., 2002; for a recent review, see Doyon et al., 2018).

The motor task was performed over three sessions. Each session was initiated with verification of the participant’s familiarity with the keypad. After successful verification, participants were asked to reproduce the target sequence accurately three times in a row to make sure that they understood the task. Participants were then instructed to generate the target sequence repeatedly “as fast and as accurately as possible.” In case of occasional errors, participants were asked “not to correct errors and instead to continue by starting from the beginning of the sequence.”

The sequence was introduced to participants using numbers from 1 to 4 (with numbers corresponding to digits as is shown in Figure 3A). During the first session, participants were trained on either sequence A (41324) or sequence B (42314) using a block design (14 blocks). The untrained sequence was later used to measure specificity of the acquired knowledge (see below). The next two sessions were used to assess participants’ performance, hence, were retested on the trained sequence 12 and 24 hours post-training by performing four blocks of the trained sequence in each session (Figure 1C).

Each block (in training and retests) consisted of 60 keypresses (equivalent to 12 repetitions of the 5-element sequence). These performance periods were separated by short periods of rest (25 sec). During rest periods a red cross was presented in the middle of the screen, and during performance blocks the cross was shown in green. The color of the cross automatically changed from red to green and from green to red indicating the beginning and the end of each block, respectively. During the task, participants were given only these visual cues and did not receive any feedback (Figure 3B).

To test generalization of the skill to the untrained hand, participants took part in three additional 4-block tests at the end of the third session. All participants performed these tasks in the same order: (a) the trained sequence (T-S) performed with the untrained hand (U-H), (b) the untrained sequence (U-S) performed with the trained hand (T-H), and (c) the untrained sequence (U-S) performed with the untrained hand (U-H). The latter two tests (i.e., performing the untrained sequence with both hands) were meant to serve as a reference to measure the specificity of the acquired knowledge to the trained sequence. Nevertheless, due to a technical error, the untrained sequence was always performed in the trained hand before the untrained hand and was thus already familiar when performed by the untrained hand. We therefore do not include this (c) condition in the analysis.

### Statistical Analyses

We analyzed the data using mixed repeated measures ANOVAs with *consolidation interval* as a within-subject factor and *group* as a between-subject factor. We also used one-sample *t* tests to estimate the magnitude of gains, and two-sample *t* tests to examine differences between groups. The distribution of measures of interest across individuals was tested for skewness and kurtosis.

Since the protocol was very demanding, including seven meetings per participant, scheduled in fixed hours of the day, requiring participants to abstain from caffeine and maintain rigorous sleep hygiene for several days, participant groups were relatively small, possibly compromising the statistical power of the study. Therefore, to examine the strength of evidence, additional Bayesian analyses were carried out in parallel to the frequentist analyses (ANOVAs and *t* tests), to assess the odds for the research and null hypotheses given the obtained data. Bayes Factors (*BF*) were computed using JASP (version 0.8.6.0; cf. 2017; van Doorn et al., 2020; Wagenmakers et al., 2018). Additionally, post hoc sensitivity analysis was computed in G*power 3.0.10 (Faul et al., 2007) in order to determine the minimum reliable effect size at 80% power level given our sample size.

#### Language Learning Task

Only consistent words were included in the analysis of trained items. Reaction times (RTs) for correct responses were calculated. Percentage of correct responses was calculated for each individual in each token frequency (high and low) at each testing point.

As our main research questions focus on the consolidation period (i.e., from the end of training to 12 and 24 hours post-training), our analyses were conducted on three measures, calculated separately for accuracy and RT of trained items and for accuracy in untrained items: (1) gains during the first 12 hours after training, (2) gains during the second 12 hours post-training, and (3) gains across the entire 24 hours from the end of training. Individual gains in each interval were calculated as a ratio from the performance level at the beginning of that interval, e.g., the *first 12-hr interval* = [(difference in performance levels from the end of training test to the 12-hr retest) / end of training test]. The *change in performance* was always calculated so that higher (more positive) values indicate improvement. Hence for RT, it was calculated as [performance at the end of training minus the 12-hr retest] while for accuracy it was calculated as [performance at the 12-hr retest minus performance at the end of training]. Similarly, the *second 12-hr interval* = [(difference in performance levels from the 12-hr to the 24-hr retest) / the 12-hr retest]. Finally, the *total 24-hr interval* was calculated as [(difference in performance levels from the end of the training test to the 24-hr retest) / the end of training test]. Normalized gains in performance were used in all analyses to control for individual differences in absolute performance levels and allow for a between groups comparison, as was used in previous studies exploring the effect of consolidation on offline gains (Abend et al., 2013; Doyon et al., 2009; Korman et al., 2007, 2021). For each measure we first examined whether participants improved in performance during the total 24-hr consolidation period (this was done across groups when there was no difference between groups). We then examined the effect of sleep on the first and second 12-hr intervals by comparing between intervals and groups.

Participants’ explicit awareness of the morphophonological rule as expressed in the questionnaire was scored on a scale of 1 (no knowledge or uncalculated guess) to 6 (precise explicit regularity knowledge). (See Table 3 for scoring criteria.) Participants’ awareness score in each group was estimated against the score of “2,” which represents a non-phonological strategy, using a one-sample *t* test. These scores were also compared between groups using a two-sample *t* test. The correlation of explicit knowledge with raw performance and consolidation (both 12-hr intervals and the total 24-hr interval) on the language task was tested using Spearman correlations.

**Table 3.** Scoring criteria of awareness to the morphophonological regularity

**Score**	**Level of awareness**
**1**	Uncalculated guess
**2**	Non-phonological strategy (e.g., semantic)
**3**	Basic phonological strategy leading to knowledge of one suffix (VC) or consonant with suffix (CVC)
**4**	Intermediate phonological strategy leading to two suffixes (VC) or consonant with suffixes (CVC)
**5**	Phonological strategy leading to three suffixes (VC) and consonant with suffixes (CVC)
**6**	Full phonological strategy leading only to three distinguished suffixes (VC)

#### Motor Learning Task

Motor skill was evaluated using a measure reflecting the tapping speed, namely the time (duration) to complete each block (i.e., 60 key presses). The percentage of correctly performed and completed sequences, out of 12 possible sequences per block, was also calculated (accuracy). At each time point, performance was calculated as the mean across four blocks. The normalized consolidation intervals and the statistical analysis of the trained sequence were calculated as in the language learning task, as was the analysis relating to the change in performance across the total consolidation interval and the effect of sleep on consolidation.

To test whether the motor skill was generalized to new conditions, we tested the specificity of the skill to the trained sequence in comparison to a new sequence, and its transfer to the untrained hand at the end of the third session (at the 24-hr retest). *Sequence specificity* in the trained hand and *transfer* of the trained sequence to the untrained hand were computed for each individual, based on the average time-per-block of the 4 blocks, measured at the 24-hr time point, and normalized to the individual’s performance in order to account for general individual differences in speed. Thus, *Sequence Specificity* = [(Untrained Sequence − Trained Sequence) / Untrained Sequence]. Higher (positive) values reflect higher specificity (i.e., better performance of the trained than the untrained sequence). *Transfer to the untrained hand* = [(Trained Sequence in the untrained hand – Trained Sequence in the trained hand at the 24-hr retest) / Trained Sequence in the untrained hand]. Smaller values reflect better transfer (i.e., smaller differences between the trained and untrained hand). Both measures were compared to zero within each group using a one-sample *t* test and compared between groups using a two-sample *t* test.

### Correlation Analyses

To examine the extent to which performance on trained items was related to performance on untrained items, in both linguistic and motor tasks, we conducted a correlation analysis within each task using both raw performance and normalized consolidation intervals. Correlation analyses were also conducted for individual consolidation gains between the linguistic and motor tasks. The results were corrected for multiple comparisons using Bonferroni correction.

## RESULTS

### Group Effect on Consolidation

#### Language learning task

##### Trained items.

**Figure 4.**
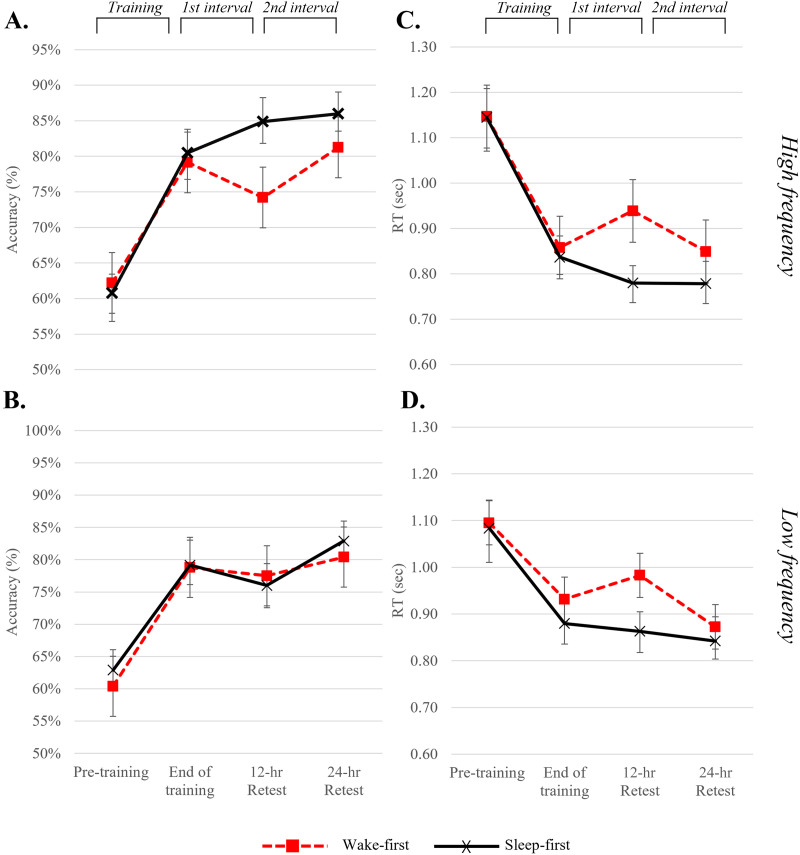
Raw performance on trained items at four testing points, presented by frequency and group. (A) and (B) Accuracy rates and (C) and (D) reaction times (RT) for the high frequency (upper panels) and low frequency (lower panels). This figure, showing raw performance, is presented for display purposes only, as the statistical analyses were conducted on normalized consolidation intervals.

**Figure 5.**
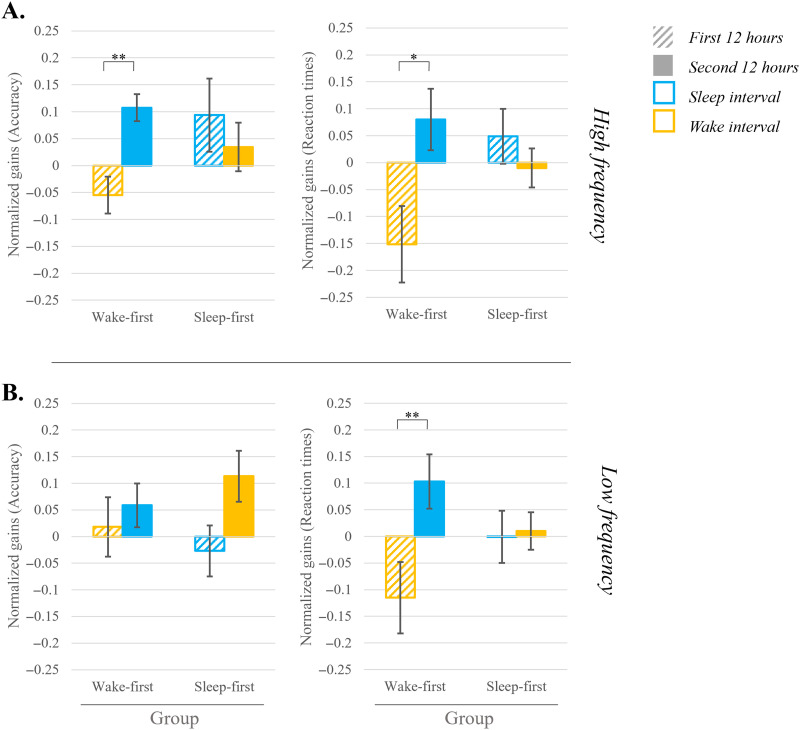
Gains in performance in the language task, presented by frequency and group (wake-first/sleep-first). (A) High frequency and (B) Low frequency. 12-hr gains are presented for the two consolidation intervals (first 12 hours striped bar; second 12 hours full bar) with the color indicating if an interval includes sleep (blue) or wake (yellow). Accuracy rate is presented on the left side and reaction times on the right. Error bars indicate standard error. For both measures positive values indicate improvement. *indicates significance at *p* < 0.05; **indicates significance at *p* < 0.001.

Figure 4 shows the raw performance on high and low frequency trained items for descriptive purposes. Nevertheless, all statistical analyses were conducted on normalized individual consolidation gains (as described in the Materials and Methods section), shown in Figure 5.

###### Consolidation across 24 hours.

Our first research question was whether participants improved during the consolidation period from the end of training to the 24-hr retest, and whether this was affected by group or item frequency. To this end we conducted a two-way repeated measures ANOVA on the total consolidation gain in accuracy with frequency (high and low) as a within-subject factor and group (wake-first, sleep-first) as a between-subject factor. There were no significant main effects or interactions and the *BF*s showed moderate to strong support for H_0_ (0.087 ≤ *BF*_incl_ ≤ 0.247). We thus collapsed the gains in the high and low frequency items, and combined the two groups, and tested whether the 24-hr consolidation gain across item frequencies and groups was significantly different from zero in a one-sample *t* test. This analysis revealed an overall small improvement across 24 hours [*t*(37) = 2.556; *p* = 0.015, *d* = 0.414; *BF*_10_ = 2.974 (anecdotal support for H_1_)]. These analyses were also conducted for RTs, finding the same pattern of no difference between groups and frequencies in RT gains across 24 hours, with strong to anecdotal support for H_0_ (0.096 ≤ *BF*_incl_ ≤ 0.363). The gains in RTs during the 24-hr consolidation period were not significantly different from zero [*t*(37) = 114; *p* = 0.910, *d* = 0.018; *BF*_10_ = 0.176 (moderate support for H_0_)], suggesting stable performance across 24 hours. Hence, across 24 hours post-training participants showed some improvement in accuracy levels and stable reaction times, both of which did not differ across frequencies and groups.

###### The effect of sleep on consolidation.

To probe for the effect of *sleep* on consolidation, we performed a three-way repeated measures ANOVA on 12-hr consolidation gains, with interval (first, second) and frequency (high and low) as within-subject factors and sleep group (wake-first vs. sleep-first) as a between-subject factor. For *reaction times*, we found a significant interaction between interval and sleep group [*F*(1, 36) = 9.056; *p* = 0.005; η^2^ = 0.201; *BF*_incl_ = 42.735 (very strong support for H_1_)], indicating that sleep affects consolidation of trained items across item frequencies. The three-way interaction between frequency, interval, and sleep group was not significant [*F*(1, 36) = 0.335; *p* = 0.566; η^2^ = 0.009; *BF*_incl_ = 0.059 (strong support for H_0_)]. For *accuracy*, this analysis revealed a marginally significant three-way interaction between frequency, interval, and sleep group [*F*(1, 36) = 4.031; *p* = 0.052; η^2^ = 0.101; *BF*_incl_ = 0.128 (moderate support for H_0_)]. Because we had distinct theoretical predictions for the high and low frequency items, which are expected to rely to some extent on different mechanisms, we wanted to examine whether the effect of sleep was due to one or both frequency levels. Therefore, although the three-way interaction with frequency was only marginal for accuracy, we conducted follow-up analyses separately for each frequency, for both accuracy and RTs.

For high frequency trained items a two-way repeated measures ANOVA on consolidation gains in *accuracy* comparing the first and second 12-hr intervals, and the two sleep groups, revealed no main effects of either interval (*BF*_incl_ = 0.670, anecdotal support for H_0_) or group (*BF*_incl_ = 0.546, anecdotal support for H_0_). However, the *interaction between interval and group* was significant [*F*(1, 36) = 4.344; *p* = 0.044; η^2^ = 0.108; *BF*_incl_ = 1.445 (anecdotal support for H_1_)]. To follow-up on this interaction, we conducted a paired-sample *t* test comparing *consolidation intervals* within each *group*. Participants in the wake-first group showed a significant difference between *intervals* [*t*(19) = −3.364; *p* = 0.003; *d* = 0.752; *BF*_10_ = 13.191 (strong support for H_1_)]. Figure 5A (left panel) shows that this was due to deterioration during wake and improvement during sleep. In contrast, in the sleep-first group there was no difference between intervals [*t*(17) = 0.602; *p* = 0.555; *d* = 0.141; *BF*_10_ = 0.286 (moderate support for H_0_)]. Figure 5A shows that when sleep occurred in the first interval, performance was stable during the subsequent wake interval.

The two-way repeated measures ANOVA performed on consolidation gains in *reaction times* (first and second 12-hr intervals) showed no significant main effects of either interval (*BF*_incl_ = 1.404, anecdotal support for H_1_) or group (*BF*_incl_ = 0.901, anecdotal support for H_0_), but again revealed a significant *interaction between interval and group* [*F*(1, 36) = 5.198; *p* = 0.029; η^2^ = 0.126; *BF*_incl_ = 2.958 (anecdotal support for H_1_)]. A follow-up analysis on this interaction, conducted separately for each group using a paired-sample *t* test, revealed, once again, a significant difference between *intervals* for the wake-first group only [*t*(19) = −2.307; *p* = 0.032; *d* = −.516; *BF*_10_ = 1.956 (anecdotal support for H_1_)]. Here as well, observation of Figure 5A (right panel) shows that this was driven by deterioration in performance during wake and an improvement during sleep. No significant difference between the intervals was found for the sleep-first group [*t*(17) = 0.788; *p* = 0.442; *d* = 0.185; *BF*_10_ = 0.320 (moderate support for H_0_)]. Figure 5A shows again that when sleep occurred during the first interval, performance was stable during the subsequent wake interval.

For low frequency trained items a two-way repeated measures ANOVA performed on consolidation gains in *accuracy* (first 12 hours and second 12 hours) revealed no significant main effects of interval or group, nor an interaction (and the *BF* showed moderate to anecdotal support for H_0_ (0.191 ≤ *BF*_incl_ ≤ 0.726). However, for *reaction times* the two-way repeated measures ANOVA performed on consolidation gains (first 12 hours and second 12 hours) revealed a significant *main effect of interval* [*F*(1, 36) = 6.731; *p* = 0.014; η^2^ = 0.158; *BF*_incl_ = 5.535 (moderate support for H_1_)] with no main effect of group (*BF*_incl_ = 0.723, anecdotal support for H_0_). This analysis also showed a *significant interaction between interval and group* [*F*(1, 36) = 5.488; p = 0.025; η^2^ = 0.132; *BF*_incl_ = 2.167 (anecdotal support for H_1_)]. Follow-up analyses, using paired-sample *t* tests to compare between the *consolidation intervals* within each group, revealed a significant difference between *intervals* for the wake-first group [*t*(19) = −3.541; *p* = 0.002; *d* = 0.791; *BF*_10_ = 18.630 (strong support for H_1_)]. Figure 5B (right panel) shows that this is driven by deterioration during wake and improvement during sleep. No significant difference between the intervals was found for the sleep-first group [*t*(17) = −0.176; *p* = 0.862; *d* = 0.041; *BF*_10_ = 0.246 (moderate support for H_0_)]. Figure 5B shows that when sleep occurred in the first interval performance was stable across the first and second intervals.

##### Untrained items.

**Figure 6.**
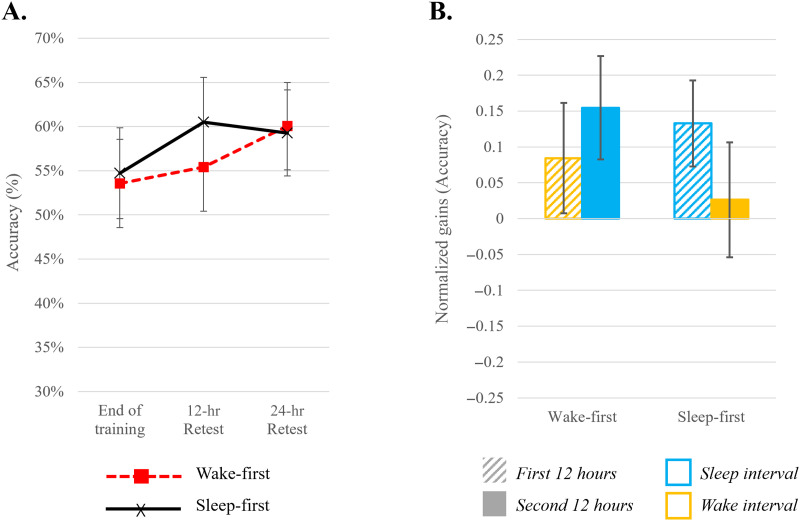
(A) Raw performance accuracy of untrained items, by group (wake-first/sleep-first) presented at three testing time points. The dashed line at 33.3% represents chance level. (B) Gains in performance for untrained items, presented by group: 12-hr gains are presented for the two consolidation intervals (first 12 hours striped bar; second 12 hours full bar) with the color indicating if an interval includes sleep (blue) or wake (yellow). Error bars indicate standard errors.

Figure 6A shows the raw performance on untrained items for the three testing time points. Preliminary analyses on raw performance using one-sample *t* tests showed that participants performed above chance level (33.3%) at all testing time points (5.93 ≤ *t* ≤ 7.803, all *p* < 0.001, corrected for 3 time points; see Figure 6A). All subsequent statistical analyses were conducted on normalized individual gains (shown in Figure 6B).

An independent-sample *t* test on the 24-hr interval showed no significant difference between groups [*t*(36) = 0.789; *p* = 0.436; *d* = 0.26; *BF*_10_ = 0.391 (anecdotal support for H_0_)]. Therefore, to examine the pattern of change during the 24-hr consolidation period, we collapsed across both groups and tested whether the 24-hr gain was different from zero using a one-sample *t* test. We found a small and significant improvement in performance across groups during the 24-hr consolidation period [*t*(37) = 2.445; *p* = 0.019; *d* = 0.396; *BF*_10_ = 2.407 (anecdotal support for H_1_)]. To address the question of the effect of sleep on consolidation, a two-way repeated measures ANOVA on the 12-hr intervals (first and second 12 hours) revealed no significant main effects of interval or group and no interaction, (all *BF* showed moderate support for H_0_ (0.106 ≤ *BF*_incl_ ≤ 0.220). See Figure 6B.

#### Motor task.

**Figure 7.**
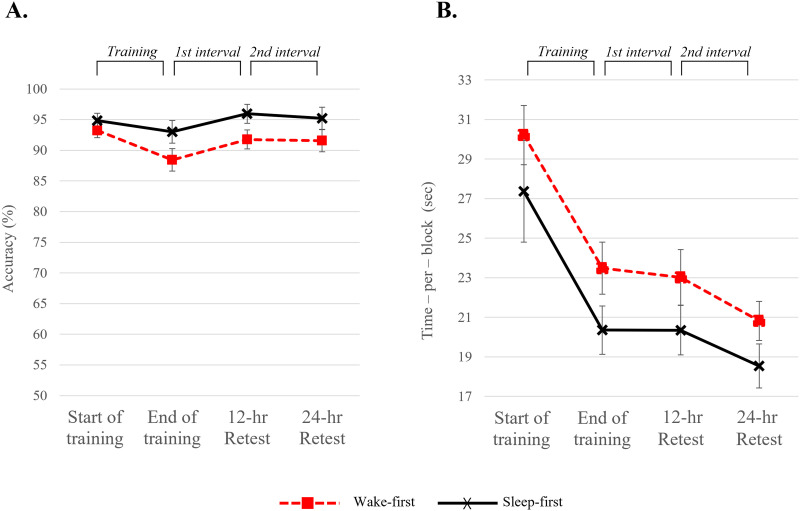
Performance on the trained motor sequence presented by group, both for (A) Accuracy and (B) Time per block. Each time point represents the mean values of four blocks of 60 key-presses each. This figure of raw performance is presented for display purposes only, as the statistical analyses were conducted on normalized consolidation intervals.

**Figure 8.**
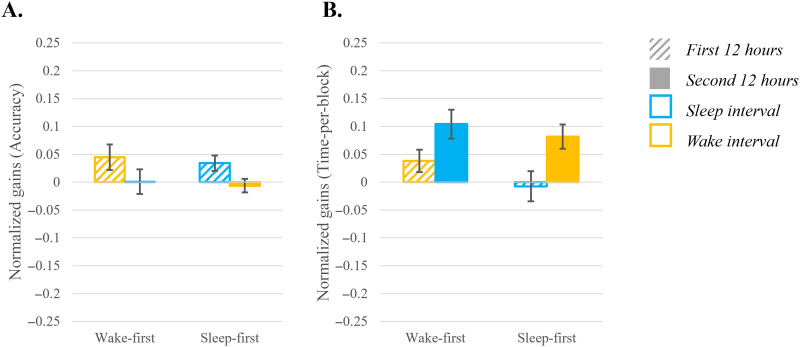
Gains in performance in the motor task, in Accuracy (A) and Time-per-block (B), presented for the two consolidation intervals (first 12 hours striped bar; second 12 hours full bar) with the color indicating if an interval includes sleep (blue) or wake (yellow). Error bars indicate standard error. In both Accuracy and Time-per-block positive values indicate improvement.

Figure 7 presents the raw performance in the motor task for descriptive purposes. All statistical analyses were conducted on normalized individual intervals (see Figure 8).

##### Trained sequence.

###### Accuracy.

Participants’ performance was highly *accurate* with 92.86% ± 4.95% (mean ± *SEM*) accuracy on average throughout the experiment, consistent with previous studies on motor sequence learning (Gabitov et al., 2014, 2017; Korman et al., 2003; Walker et al., 2002). An independent-sample *t* test on the 24-hr interval showed no significant difference between groups [*t*(34) = 0.623; *p* = 0.537; *d* = 0.21; *BF*_10_ = 0.375 (anecdotal support for H_0_)]. We therefore collapsed across groups and used a one-sample *t* test to test whether the overall change in performance over 24 hours was different from zero. There was a significant improvement during the 24-hr consolidation period [*t*(35) = 2.746; *p* = 0.009; *d* = 0.457; *BF*_10_ = 4.432 (moderate support for H_1_)]. To test the effect of sleep on consolidation a two-way repeated measures ANOVA performed on the 12-hr intervals (first and second 12 hours) revealed *a marginal main effect of interval* [*F*(1, 34) = 3.996; *p* = 0.054; η^2^ = 0.105; *BF*_incl_ = 9.248 (moderate support for H_1_)] driven by greater improvement during the first 12 hours and stabilization during the second 12 hours, across groups. There was no main effect for group (*BF*_incl_ = 0.071; strong support for H_0_) nor an interaction between interval and group (*BF*_incl_ = 0.083; strong support for H_0_) (see Figure 8A).

###### Time per block.

As performance accuracy is typically very high in this task (Gabitov et al., 2014, 2017; Korman et al., 2003; Walker et al., 2002), the main learning outcome measure is *speed* measured as *time per block*. An independent-sample *t* test on the 24-hr interval showed no significant difference between groups [*t*(34) = 0.585; *p* = 0.563; *d* = 0.20; *BF*_10_ = 0.368 (anecdotal support for H_0_)]. Hence, we collapsed across groups to examine the improvement across 24 hours. A significant and very strong improvement in performance was found across 24 hours [*t*(35) = 4.917; *p* < 0.001; *d* = 0.891; *BF*_10_ = 1053.055 (extreme support for H_1_)].

In order to test the effect of sleep on consolidation, a two-way repeated measures ANOVA on the 12-hr intervals was conducted, with *consolidation intervals* as a within-subject factor and *group* as a between-subject factor. The analysis revealed a significant *main effect for consolidation interval* [*F*(1, 34) = 4.945; *p* = 0.033; η^2^ = 0.127; *BF*_incl_ = 3.222 (moderate support for H_1_)] indicating stable performance during the first interval and subsequent improvement during the second interval across groups. No significant effect was found for *group* (*BF*_incl_ = 0.378, anecdotal support for H_0_) nor *an interaction* between *interval* and *group* (*BF*_incl_ = 0.761, anecdotal support for H_0_), indicating that offline gains appeared only 24 hours post-training, independent of the timing of sleep (see Figure 8B).

###### Generalization.

The specificity of participants’ skill to the trained sequence, as measured by the normalized *sequence specificity* index 24 hours post-training, was not different between the groups as was indicated by an independent-sample *t* test [*t*(34) = 0.198; *p* = 0.884; *d* = 0.06; *BF*_10_ = 0.327 (moderate support for H_0_)]. This indicates that the proximity of the training session to sleep did not affect sequence specificity 24 hours post-training. We therefore collapsed across groups, and found that sequence specificity was significantly above zero [*t*(35) = 6.341, *p* < 0.001; *d* = 1.05; *BF*_10_ = 57587.32 (extreme support for H_1_)], indicating that the trained sequence was performed better than the untrained sequence.

Participants’ generalization of gains to the untrained hand, as measured by the normalized *transfer to untrained hand,* were not different between groups [*t*(34) = 1.630; *p* = 0.122; *d* = 0.55; *BF*_10_ = 0.893 (anecdotal support for H_0_)]. We therefore collapsed across groups and using a one-sample t test found that this measure was not significantly different from zero [*t*(35) = 1.585; *p* = 0.122; *d* = 0.26; *BF*_10_ = 0.559 (anecdotal support for H_0_)], indicating no difference between the trained and untrained hands.

### Individual Differences in Consolidation

#### Correlations within the language domain

To examine the extent to which performance on trained items (which can rely on item-specific learning and on extraction of regularities) depends on extraction of regularities (measured by performance on untrained items), we calculated Pearson correlations between raw *accuracy* levels on the trained and untrained items at each testing time point across groups, separately for low and high frequency trained items. These analyses revealed strong and significant positive correlations between tasks for all 3 testing time points (0.486 ≤ *r* ≤ 0.713, all *p* ≤ 0.002, corrected for 6 comparisons). Hence, better ability to recall high and low frequency trained items was positively associated with the ability to extract the phonological regularities and implement them to untrained items immediately post-training, and throughout the other tests. However, when examining the extent to which the consolidation of extraction of regularities is related to the consolidation of trained items, by testing for correlations between normalized consolidation intervals of the trained and untrained items (both 12-hr intervals and the total 24-hr interval), no significant correlations were found.

To test whether participants developed explicit awareness of the morphophonological regularities by the end of the experiment, and whether this was associated with their consolidation in the language task, we used the results of the questionnaire administered at the end of the third session. Participants from both groups developed a basic level of awareness of the morphophonological regularities, as indicated by mean awareness of 2.88 (± 1.56) for the wake-first group and 3.611 (± 1.61) for the sleep-first group. The groups did not differ from each other in the levels of awareness [*t*(34) = −1.362; *p* = 0.182; *d* = 0.46; *BF*_10_ = 0.659 (anecdotal support for H_0_)], so we collapsed across groups and using a one-sample *t* test found that this level of awareness was significantly above 2 (representing a non-phonological strategy; [*t*(35) = 4.658; *p* < 0.001; *d* = 0.776; *BF*_10_ = 516.85 (extreme support for H_1_)]. Explicit knowledge was significantly correlated (Spearman rank order correlation) with raw performance *accuracy* on trained and untrained items at all 3 testing points (0.441 < *r* < 0.658, all *p* ≤ 0.01, corrected for 3 comparisons). However, explicit regularity knowledge was not associated with gains in either of the 12-hr intervals or the total 24-hr interval, for either trained or untrained items across groups.

#### Correlations within the motor domain

To examine the association between consolidation in the motor task, and *specificity* of the acquired skill to the trained sequence, we examined the correlation between participants’ consolidation in speed across 24 hours and *sequence specificity*. Because there were no differences between groups in any of these measures, this was done across groups. The analysis revealed a marginal positive correlation between offline gains and sequence specificity in the trained hand (*r*(37) = 0.324, *p* = 0.054; *d* = 0.10), indicating that participants who improved more during the 24-hr interval tended to show more specificity to the trained sequence 24 hours post-training.

In contrast, we found no association between consolidation in the motor task across 24 hours and *transfer* of the acquired sequence-specific skill to the untrained hand (*p* = 0.074, following the exclusion of two outliers).

#### Correlations between the language and motor domains

To test whether there are individual differences in consolidation that are shared across the motor and language domains, we examined the correlations between the 24-hr interval in the two domains in terms of speed, which is the more relevant measure for the motor task. We found no significant correlation between the overall normalized gains across 24 hours in the motor and the *high frequency* trained items in the linguistic task (*r*(36) = 0.016, *p* = 0.924; *d* = 0.00). However, the 24-hr consolidation gains of the motor task were significantly correlated with the 24-hr gains in the *low frequency* trained items in the linguistic task, though the effect size was relatively small (*r*(36) = 0.329, *p* = 0.049; *d* = 0.10). Nevertheless, no significant difference was found between the high and low frequency items in terms of their correlation with the motor consolidation (*z* = −1.323; *p* = 0.093). Thus, participants who improved more across 24 hours in the motor domain also showed greater improvement in the linguistic domain, but only for the low frequency trained items.

### Sensitivity Analysis

Sensitivity analysis, computed in G*power 3.0.10 (Faul et al., 2007) with alpha = 0.05, revealed that our current sample size had 80% power to detect an effect size of *d* ≥ 0.466, for one-sample *t* tests, which were conducted in the current study in order to test for an overall improvement across 24 hours. Our findings of significant improvement over 24 hours in trained and untrained items in the language task are slightly below this effect size, which together with the *BF* suggests that these findings could be anecdotal. In contrast, the 24 hours improvement in the motor task showed a larger effect size, suggesting it is reliable. In addition, sensitivity analysis with the above parameters was also computed for a two-way repeated measures ANOVA with within-between interactions, conducted in the current study to test for the effect of sleep on consolidation. It revealed that our sample size had enough power to detect an effect size of *f* ≥ 0.234 (i.e., η^2^ ≥ 0.051). All significant interactions in the current study showed larger effect sizes, adding confidence to their reliability. Finally, sensitivity analysis with the above parameters was computed for paired-sample *t* tests, conducted in the current study for testing the differences between the first and second consolidation intervals within group. This showed there is enough power to detect an effect size of *d* ≥ 0.660. Two of the three significant findings showed larger effect sizes (high frequency accuracy, and low frequency RT) suggesting these findings have higher reliability.

## DISCUSSION

The current study examined patterns of improvement and stabilization, sleep dependence, and domain generality of memory consolidation, by comparing consolidation in a language learning task and a motor sequence learning task and the correlations between the two. The structure of the language learning task allowed us to probe two varieties of learning: memorization of exemplars, namely, item-specific learning, and the extraction of patterns embedded in the input into grammatical regularities. This distinction between components in the language task allowed us to specifically address the effects of consolidation of these components. In addition, consolidation patterns of the language task were compared to consolidation of motor sequence learning to probe for common mechanisms, both at the group level, and in terms of individual differences. Below we discuss the results in view of the current literature and in projection to wider implications.

### Consolidation in Language Learning

In the current study, we charted the consolidation and the role of sleep in learning morphological inflections over the course of 24 hours in typical adults divided into two groups, wake-first and sleep-first. In discussing the results, we address findings relating to trained items and untrained items separately, as each reflects, to some extent, distinct linguistic components.

#### Consolidation and the role of sleep in item-specific learning

Participants’ *accuracy* in inflecting all trained items showed a small improvement 24 hours after training, indicating continued offline processing of the acquired information after training. Importantly, consolidation of trained items was affected by sleep. This was shown in reaction time by the strong evidence for an interaction between interval and sleep group in both high and low frequency items. For reaction time of all trained items, while the wake-first group showed deterioration during wake and recovery in the subsequent sleep interval, the sleep-first group showed stable performance across the two intervals. Interestingly, for accuracy, the effect of sleep on consolidation was only evident in high frequency items (although it should be noted that the interaction with frequency was only marginal). For these high frequency items, the effect of sleep was similar to reaction times: The wake-first group deteriorated during wake and recovered during the subsequent sleep, while the sleep-first group showed no such deterioration during wake. Altogether these results show a clear effect of sleep on consolidation of trained items, but while high frequency items showed an effect of sleep in both accuracy and reaction time, low-frequency items show this effect only in reaction time. Because high and low frequency items share the same regularities, the differences between them in the effect of sleep on accuracy can most likely be attributed to item-specific learning, which played a more prominent role in high frequency items. In contrast, for low frequency trained items, which should rely more on the extraction of regularities, the effect of sleep on consolidation was only partial.

The stabilizing effect of sleep on consolidation of trained items was expressed in several ways. (1) In the sleep-first group, during the first interval, sleep prevented degradation of memory that was evident in the comparable interval for the wake-first group, most likely due to interference. (2) During the second interval, the sleep-first group maintained stable performance of the learned linguistic information despite being awake. Thus, sleep immediately after training *protected* against future wake interference. (3) When the wake-first group slept, during their second interval, they managed to recover their performance, which had deteriorated in the previous wake interval. Thus, sleep facilitated recovery after memory degradation.

While the first of these findings can also be explained by a model in which sleep provides only a passive protection from interference (Jenkins & Dallenbach, 1924; Mednick et al., 2011), the latter two findings (sleep protecting against later degradation and sleep facilitating recovery) argue against such a model, and suggest that sleep plays an active role in consolidation and is associated with qualitative changes to memory representations due to specific sleep-related neural processes. This hypothesis is consistent with other studies suggesting that during stabilization, memory traces are actively strengthened during sleep (Diekelmann & Born, 2010), and thus accessibility to the memories improves (Dumay, 2016). Moreover, this active information processing that occurs during sleep creates more durable memory traces, which are then more resistant to interference introduced after the sleep period, resulting in stabilization of performance (Brawn et al., 2010; Fenn et al., 2003; Sonni & Spencer, 2015). This process was also specifically suggested for consolidation of episodic memories (Ellenbogen, Payne, & Stickgold, 2006) during which spontaneous reactivation occurring in the hippocampus during sleep improves memory maintenance (Schreiner & Rasch, 2018).

Our findings, showing a stabilizing effect of sleep on trained items, and more prominent effects on trained items with better opportunities for item-specific knowledge (high frequency items), are in line with previous evidence for a stabilization effect of sleep on episodic learning in general (Ellenbogen, Hulbert et al., 2006; Schönauer et al., 2015) and specifically on vocabulary learning (Dumay, 2016; Gais et al., 2006). Given the important role of hippocampal-dependent learning in vocabulary learning (Davis & Gaskell, 2009), this suggests that the effects of sleep on learning of trained items in the current study also reflect hippocampal-based item-specific learning.

#### Consolidation and the extraction of linguistic regularities

In the current study, participants were also tested on their ability to generalize their knowledge of morphophonological regularities embedded in the trained items to the inflection of untrained items. Both groups presented above-chance performance on untrained items at the end of training and a small significant overall enhancement in performance across 24 hours, regardless of the timing of sleep. The benefit of the passage of time for regularity extraction, expressed as enhancement in performance, with no evidence that the timing of sleep influences that process, is consistent with a previous study reporting that a short nap did not enhance the extraction of grammatical regularities (Mirković & Gaskell, 2016). The current study shows no benefit of sleep even after a full night’s sleep. These findings are in contrast to some studies reporting a beneficial effect of sleep on regularity extraction through the association between sleep and grammar learning (Batterink et al., 2014; Kim & Fenn, 2020). Nevertheless, Kim and Fenn (2020) suggest that sleep benefits generalization only for learners who are aware of the regularities before sleep. In the current study, we did not measure participants’ explicit awareness of the regularities before sleep, hence variability among participants may explain this discrepancy.

The improvement in generalization during the 24-hr consolidation period is consistent with the notion that consolidation is associated with the abstraction of gist information from newly encoded memories to form cognitive schemata (Lewis & Durrant, 2011). During this process, shared information is extracted from separate exemplars resulting in the ability to generalize these commonalities to novel experiences. In the current study, the process of regularity extraction began during training, resulting in above-chance performance in the generalization test immediately after training. Interestingly, grammar knowledge continued to evolve over the next 24 hours, suggesting a qualitative change in the representations of knowledge during offline consolidation. While this grammar knowledge was not explicitly instructed, it had become conscious to some degree by 24 hours post-training, and this awareness was associated with performance on untrained items.

The association between item-specific knowledge and regularity extraction is evident in the strong positive correlations found between performance on high and low trained items on the one hand and untrained items on the other hand, throughout all testing time points. These correlations may suggest that even during training, performance on trained items was based, to some degree, on grammar knowledge. Alternatively, they may suggest that memorization of specific exemplars contributed to the evolution of grammar knowledge, consistent with evidence from computational models suggesting that improved memorization of exemplars can lead to better generalization of information (Khandelwal et al., 2019; Morcos et al., 2018).

Nevertheless, despite the above-mentioned correlations between raw performance on trained and untrained items, there were no correlations between the consolidation gains on trained and untrained items. These findings, which replicate previous studies (Ben Zion et al., 2019), may suggest that while grammar knowledge evolved from memorization of specific exemplars, the consolidation of the two types of knowledge may be mediated by distinct neurocognitive mechanisms. This finding is consistent with the idea that learning of trained items and particularly high frequency items relied mainly on item-specific memorization, which is associated with the episodic, hippocampal system and is stabilized by sleep. In contrast, the extraction of regularities may depend on frontostriatal skill learning, suggested to be involved in grammar learning (Hedenius et al., 2011; Ullman, 2015; but see also Kumaran & McClelland, 2012, for an alternative model suggesting that both exemplar memorization and generalization may be supported by the hippocampal system). Consistent with the current results, this skill-learning system may underlie offline improvement during the consolidation interval with no necessity for sleep (Kemény & Lukács, 2016; Meier & Cock, 2014; Song, 2009). Further support for this notion comes from a previous neuroimaging study that used a similar language paradigm (Nevat et al., 2017) and showed activation in the frontostriatal network during inflection of trained items.

### Consolidation in Motor Skill Learning

In the current study we also tested the role of sleep in motor sequence learning, in the same participants and with the same schedule as the language task. Thus, the sequence was trained either in the morning (wake-first group) or evening (sleep-first group) and was tested 12 and 24 hours post-training in both groups. Sequence specificity in the trained hand and transfer of the skill to the untrained hand were also tested at the 24-hr time point.

Considering speed as our main measure of interest, the results indicate that in both groups performance of the trained sequence was maintained during the first 12 hours post-training, and then improved during the next 12 hours. Thus, significant enhancement in performance was observed only 24 hours post-training regardless of the timing of sleep. These results suggest that sleep was not sufficient for consolidation to occur in the first 12 hours. Nevertheless, the total 24-hr interval included both the passage of time and sleep, for both groups. Hence, while sleep may have contributed to the evolving consolidation gains over the 24-hr interval, our design cannot test whether it was indeed necessary. The lack of overnight gains for the sleep-first group was unexpected, as many studies did show significant post sleep enhancement, in comparison to wakefulness, even following a short nap (Brawn et al., 2010; Gregory et al., 2014; Korman et al., 2007; Walker et al., 2003; Wilhelm et al., 2008). Nevertheless, a recent meta-analysis of 48 motor learning studies, out of which 23 studies used the finger-tapping paradigm, found that many of these studies did not show a significant effect of sleep on consolidation, and concluded that the effect of sleep on motor sequence consolidation is rather small (Hedges *g* = 0.47; Schmidt et al., 2020). This conclusion is in line with the results of another meta-analysis that suggests that sleep by itself is not sufficient to induce enhanced performance following motor learning (Pan & Rickard, 2015).

Regardless of the role of sleep, the enhanced performance after 24 hours in the motor task is consistent with previous motor skill learning studies showing offline improvement after the end of training (Robertson, Pascual-Leone, & Miall, 2004). Previous studies suggest that this improvement reflects a qualitative change and reorganization of motor representations during consolidation (Gabitov et al., 2014; Korman et al., 2007; Rasch et al., 2007; Tucker et al., 2006; Walker et al., 2003; for reviews, see Doyon et al., 2018; Dudai, 2004; Dudai et al., 2015). Our current findings, of a correlation between improvement in performance on the trained sequence during the 24-hr interval and sequence specificity, support this notion. Sequence specificity reflects the formation of a unified representation of the sequence as a whole from the separate representations of individual movements (Dorfberger et al., 2012). At the neural level, this transformation is supported by reorganization within the corticostriatal loop (Doyon et al., 2018), including a shift in the connectivity between the putamen and the motor cortex (Gabitov et al., 2015). Thus, our findings support the conclusion that the formation of this unified representation is associated with improved performance on the trained sequence.

Finally, our results showed similar performance levels of the trained sequence in the trained and untrained hands at the 24-hr retest. Although the untrained hand was also participants’ dominant hand, previous studies that compared the dominant and non-dominant hands before training showed no differences between the hands in initial performance levels or learning rates in the finger-tapping task (Wiestler et al., 2014) and other motor learning tasks (Panzer et al., 2009; Schambra et al., 2011). Hence, the similarity in performance between the hands at the 24-hr retest suggests that the representation of the trained skill was transferred to the untrained hand. Due to technical problems, we could not properly assess sequence specificity in the untrained hand. Nevertheless, these results are in line with previous studies showing generalization from the trained to the untrained hand (Dorfberger et al., 2012; Korman et al., 2003; Rozanov et al., 2010; Witt et al., 2010) indicating that the neural representation of the skill includes cortical areas controlling the opposite effector (Dorfberger et al., 2012; Gabitov et al., 2016).

### Shared and Distinct Consolidation Mechanisms Across Domains

The design of the current study, including the motor and language learning tasks in the same population, allowed us to examine the effect of sleep on consolidation across domains, and probe the existence of shared mechanisms both at the group and at the individual level.

At the group level, sleep played a stabilizing role in the consolidation of item-specific knowledge, but the consolidation of grammar knowledge (evident by generalization to untrained items) showed similarities to the consolidation of motor sequences. In both motor and grammar learning, performance improved 24 hours after training, and sleep was not sufficient to induce enhancement 12 hours post-training. These findings are in line with the notion that offline enhancement in skill learning is independent of the timing of sleep (Kemény & Lukács, 2016; Meier & Cock, 2014; Song, 2009). Additionally, in both domains, consolidation was associated with a qualitative change to the representation. In the language task, this was evident directly in the increase in generalization during the consolidation interval, indicating that the knowledge acquired about trained items became more abstract and generalizable. In the motor task, this was evident in the correlation between sequence specificity and consolidation of the trained sequence after 24 hours. In both cases, consolidation was associated with a process by which a sequence of separate events (cue and suffix, or separate digit movements) was transformed into a unified representation.

At the individual level, we examined the correlations between consolidation in the two tasks to identify shared neurocognitive learning mechanisms. This analysis showed a positive correlation between participants’ improvement over 24 hours in the motor task and their 24-hr consolidation in the low frequency trained items in the linguistic task, indicating that some aspects of consolidation are domain general, and may characterize individuals regardless of the specific skill being learned. Another interesting aspect of this correlation is the finding that it was evident only for low (and not high) frequency items in the linguistic task (though the difference between the correlations was not significant). Given that learning of low-frequency trained items relies on regularity extraction, more than the high-frequency items, this correlation may be due to the skill learning component in both motor and grammar learning. One caveat to this conclusion is that we did not find a correlation between consolidation in the motor task and consolidation of untrained items in the linguistic task, which also relies on grammar learning.

Taken together, our results at the group and individual levels suggest that some aspects of consolidation are shared across the motor and language domains, and more specifically between motor sequence learning and grammar. This conclusion is in line with the idea that motor sequence and grammar learning recruit overlapping neural substrates (Cona & Semenza, 2017; Ferstl et al., 2008; Hertrich et al., 2016; Nakai et al., 1999) and rely on procedural/skill-learning mechanisms (Ullman & Pierpont, 2005). This suggestion earns additional support from previous findings demonstrating that consolidation in the motor and language domains is sensitive to individual differences between learners in a wide range of abilities (Adi-Japha et al., 2011; Fenn & Hambrick, 2012, 2015; Needle et al., 2015). They are also in line with the suggestion of shared consolidation mechanisms between other distinctive domains such as perceptual and motor learning (Censor et al., 2012). However, several previous studies have failed to find evidence for shared mechanisms in consolidation in the language and motor domains (Henderson & Warmington, 2017; Wilson et al., 2012). We suggest that this divergence across studies might be a consequence of the specific tasks selected for each domain in previous research (Wilson et al., 2012), or of the consolidation intervals selected (Henderson & Warmington, 2017). This fascinating issue undoubtedly deserves continued systematic research.

### Limitations

The current study should be viewed considering several limitations. First, due to the very demanding protocol, the number of participants in the study was relatively small (*N* = 38). Thus, to distinguish between underpowered and more reliable findings, we also included the results of the Bayes factor analyses and post hoc sensitivity analysis (at 80% power). Future research should aim to replicate the results with larger sample sizes, possibly using power analysis before data collection (as elaborated in Brysbaert, 2019). Second, the design of the motor task in the current study did not allow us to identify the association between consolidation and generalization of sequence specific knowledge to the untrained hand. This is because the untrained sequence was introduced in a fixed order, first to the trained and then to the untrained hand. This resulted in familiarity with this sequence when performed by the untrained hand, so it could no longer serve as a baseline for sequence specificity. Future studies can use a unique untrained sequence for each hand, which would allow proper testing of the ability to apply a newly learned order of movements to a new condition. Third, our study was designed to test the effect of sleep in intervals of 12 hours. The finding that motor sequence learning and linguistic regularity extraction were enhanced across 24 hours, which includes sleep in both groups, does not enable us to examine the contribution of sleep to consolidation in a 24-hour interval. Therefore, it is possible that sleep alone or time alone are not sufficient for offline gains, and both may be needed for consolidation. This question would require a sleep deprivation manipulation. Fourth, the smaller number of low frequency items compared to high frequency items included in the analysis raises a question of whether differences in the statistical power between high and low frequency items might explain the different effect of sleep on consolidation in the accuracy measures in these conditions. Fifth, we chose to test participants’ explicit knowledge of the regularities only at the end of the experiment, in order to minimize the potential effect of the questionnaire on the performance in the tests. As a result, we do not know the degree of explicit awareness of the regularities at the end of training, which may have affected its consolidation, as suggested by Kim and Fenn (2020). Finally, as in most studies measuring consolidation, it should be noted that offline improvement can be a result of the effect of testing, rather than a truly offline process (Eriksson et al., 2011; Schoch et al., 2017). Studies aiming to ameliorate this problem by including separate groups tested in different intervals show the benefit of active consolidation in addition to the facilitative effect of testing itself (Kroneisen & Kuepper-Tetzel, 2021).

### Conclusions

By examining the role of sleep in consolidation during grammar learning in a novel language, and comparing it to motor sequence learning, the current study was able to dissociate two patterns of consolidation in the language learning task, and find commonalities between consolidation in the language and motor domains. One component we identified is item-specific learning, which is mostly evident here in high-frequency trained items. This item-specific learning, which is similar to new vocabulary learning and relies on hippocampal mechanisms, shows active dependency on sleep that promotes its memory stabilization. The second component of language learning is the extraction of regularities embedded in the trained input. This component starts to emerge during training and continues to evolve during the consolidation period regardless of the timing of sleep. This grammar learning component, which may depend on frontostriatal skill-learning mechanisms, was similar to motor skill learning and as such reflects a commonality across domains in consolidation of skill. These similarities on the group level were further reinforced by cross-domain correlations at the individual level.

## ACKNOWLEDGMENTS

We acknowledge the data collection team and specifically Palzur Alon, Hagag Adi Anna, and Geskin Kate, research assistants, for coordinating the experiments. This work was supported by the National Institute for Neurobiology in Israel (NiPi) grant no. 208-11-12 and the Israel Science Foundation (ISF) grant no. 1052/16 to Tali Bitan.

## FUNDING INFORMATION

Tali Bitan, National Institute for Neurobiology in Israel (NiPi), Award ID: 208-11-12. Tali Bitan, Israel Science Foundation, Award ID: 1052/16.

## AUTHOR CONTRIBUTIONS

**Dafna Ben-Zion**: Conceptualization: Lead; Data curation: Lead; Formal analysis: Lead; Funding acquisition: Equal; Investigation: Lead; Methodology: Equal; Project administration: Lead; Resources: Lead; Supervision: Lead; Visualization: Lead; Writing – original & review: Lead. **Ella Gabitov**: Data curation: Support; Software: Support. *Anat Prior: Conceptualization: Equal; Methodology: Equal; Supervision: Equal; Visualization: Equal; Writing – original & review: Equal. *Tali Bitan: Conceptualization: Equal; Funding acquisition: Lead; Methodology: Equal; Supervision: Equal; Visualization: Equal; Writing – original & review: Equal. (*Equal contribution.)

## TECHNICAL TERMS

Consolidation: The process by which new and initially labile memories transform into long-term memories.
Inflectional Morphology: The process by which the grammatical category of words is changed, for example, by adding affixes.
Item-specific learning: The memorization of exemplars as whole units.
Phonology: The sound system of a language.
Token frequency: The frequency with which a whole word (token) appears in the language.
